# Xeropthalmia and optic neuropathy secondary to ARFID: a case report

**DOI:** 10.1186/s40337-024-01042-8

**Published:** 2024-07-01

**Authors:** Aletheia ZH Chia, Lim Su Ann, Bryan Sim, Courtney Davis

**Affiliations:** 1 Department of Paediatric Medicine, KK Women’s and Children’s Hospital, 100 Bukit Timah Road, Singapore, 229899 Singapore; 2 SingHealth Duke-NUS Paediatric Academic Clinical Programme, Singapore, Singapore; 3 Department of Ophthalmology, Tan Tock Seng Hospital, Singapore, Singapore; 4 Myopia Service, Singapore National Eye Centre, Singapore, Singapore; 5 Adolescent Medicine Service, Department of Paediatric Medicine, KK Women’s and Children’s Hospital, Singapore, Singapore

**Keywords:** Avoidant/restrictive food intake disorder (ARFID), Vitamin A, Vitamin deficiency, Optic neuropathy, Xeropthalmia, Case report

## Abstract

**Background:**

Patients with avoidant/restrictive food intake disorder (ARFID) commonly present with loss of weight or faltering growth in the setting of poor nutrition. However, patients with ARFID can present with micronutrient deficiencies without weight loss. In patients with ARFID, clinicians should be vigilant for micronutrient deficiencies and their presentations.

**Case presentation:**

We report a unique case of ARFID in a twelve-year-old girl, who developed micronutrient deficiencies and presented with acute visual loss with a preceding history of impaired night vision. Ophthalmic examination revealed xerophthalmia and bilateral optic neuropathy. Investigations showed severe Vitamin A and folate deficiencies which accounted for her clinical findings. In addition, she was also found to have low Vitamin B12, copper, and Vitamin D levels. She had a history of selective eating from a young age with a diet consisting largely of carbohydrates, with no regular intake of meat, dairy, fruit and vegetables. This was not driven by weight or body image concerns. The patient’s symptoms improved significantly with appropriate vitamin replacement and continued multidisciplinary care.

**Conclusions:**

This report describes a patient with ARFID presenting with visual complaints. In this case, the selective eating behaviours resulted in xeropthalmia and optic neuropathy. Micronutrient deficiencies are uncommon in developed countries. When these deficiencies are suspected, eating disorders, such as ARFID, should be considered. Similarly, clinicians caring for patients with restrictive eating disorders including ARFID should be familiar with the clinical presentations of various micronutrient deficiencies and consider evaluation and treatment for micronutrient deficiencies when clinically indicated.

## Background

Avoidant/restrictive food intake disorder (ARFID) is an eating or feeding disturbance, defined by the persistent failure to meet appropriate nutrition and/or energy needs [1]. Given its clinical heterogeneity and new inclusion into the 5th edition of the Diagnostic and Statistical Manual of Mental Disorders (DSM-5), evidence regarding the varied clinical presentations and complications of ARFID remains limited [2]. Restrictive eating in ARFID can result in significant weight loss or a failure to gain weight, growth compromise, and/or a marked interference with psychosocial functioning. Apart from reduced energy and protein intake, restrictive eating in ARFID can result in reduced micronutrient intake such as that of Vitamin B1, B2, C, K, zinc, iron and potassium [3]. Such deficiencies can have wide-ranging multi-systemic effects on health and functioning, ranging from micronutrient-specific syndromes to suboptimal growth and development.

This paper highlights the case of a 12-year-old girl with ARFID, who presented with xeropthalmia and optic neuropathy secondary to nutritional deficiencies.

## Case presentation

### Patient information

The patient was a 12-year-old female referred to the Adolescent Medicine Service for a suspected eating disorder resulting in xeropthalmia and optic neuropathy.

She was admitted to a paediatric hospital for acute, painless loss of vision in her left eye noted on awakening. On further questioning, she also reported difficulty with night vision for the past week. Clinically, she had a history of mild eczema, with exacerbation of her eczema two years prior to presentation for which she consulted a dermatologist. Despite treatment with topical steroids, the eczema did not improve. She had no other significant past medical history.

### Clinical findings

**Fig. 1:**
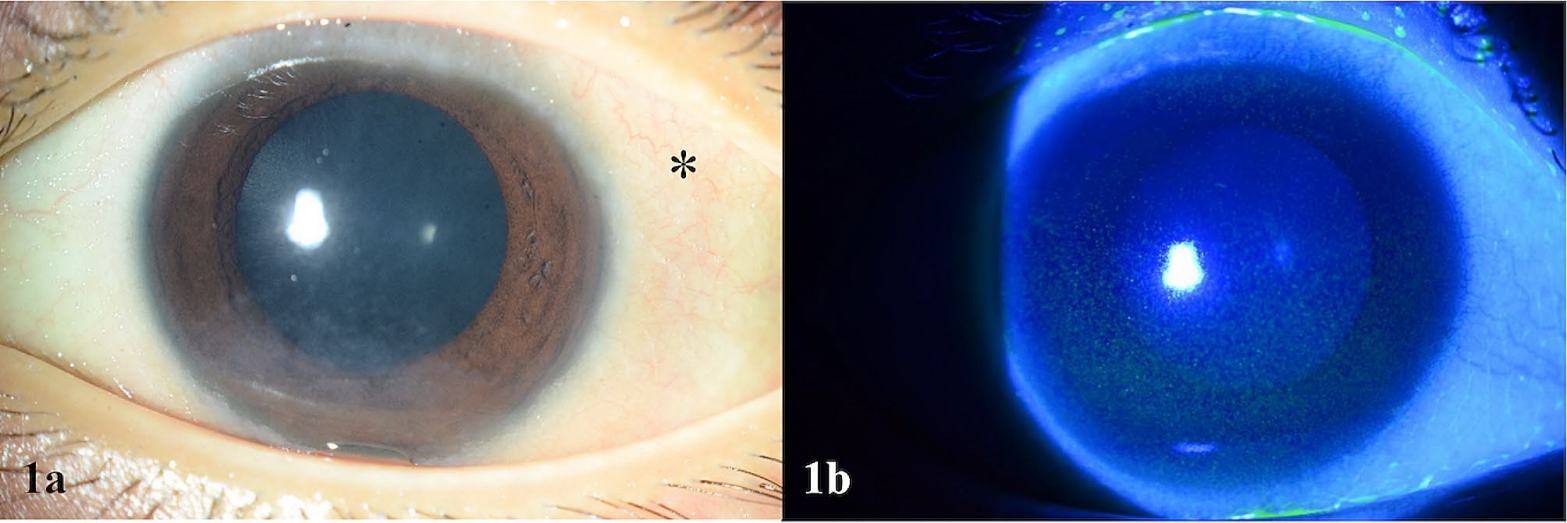
**a**: Colour photograph of the anterior segment showing xerophthalmia as evidenced by the Bitot’s spots (as marked by asterisk), collections of keratinized epithelium forming patches of foamy whitish opaque deposits, giving a shiny irregular reflex on the conjunctiva. **b**: Colour photograph of the cornea taken using cobalt blue light after fluorescein staining. Epithelial defects stain yellow. This photograph shows the multiple punctate epithelial erosions, more extensive inferiorly, due to severe dry eye

**Fig. 2:**
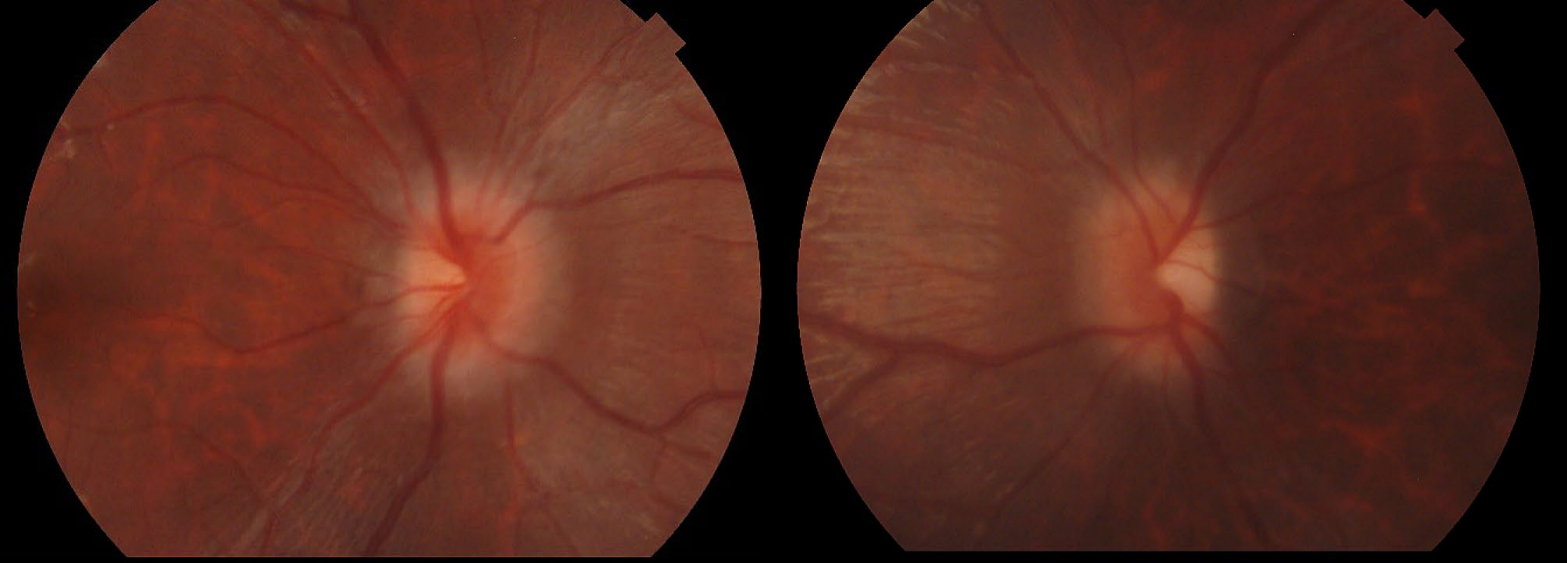
Fundal photographs showing bilateral disc swelling

At presentation, she was effectively blind in her left eye. Her visual acuity in the left eye was that of no light perception. Examination of her right eye revealed visual acuity of 6/45, impaired colour vision (she could only see the test plate on Ishihara testing) and constricted visual field both by confrontation and formal visual field testing. Anterior segment examination showed bilateral xeropthalmia with Bitot’s spots and a poor ocular surface evidenced by severe punctate epithelial erosion bilaterally, characteristic of Vitamin A deficiency (Fig. 1). Dilated fundus examination revealed bilateral optic disc swelling. (Fig. 2). Nutritional optic neuropathy related to folate, thiamine, Vitamin B12 or copper was suspected. Neurological examination was otherwise normal.

Her height was 139 cm (3rd -10th percentile) and weight was 36.4 kg (25-50th percentile) with a BMI of 18.6 kg/m^2^ (50-75th percentile). There was no significant weight loss, with stable weight and BMI percentiles over time. Menarche had occurred at the age of 11 years, with regular periods.

### Assessment

On further history, the patient was noted to have a restrictive eating pattern. Her family described her as a picky eater since early primary school when she started selecting her own food, with increasing selectiveness over the past two years. Her diet consisted primarily of carbohydrates such as bread, rice, rice porridge, and potato chips with minimal intake of vegetables or protein. She had also further restricted her food variety in the last three months after a schoolmate called her fat, and was taking bread with Nutella, a sweetened nut spread, for all meals. Although her food choice had become more limited, the portion size and estimated calorie intake had remained stable. There was no documented history of weight loss.

She shared that she disliked spicy food, meat, and vegetables, but denied any specific textural or colour preferences. She otherwise denied significant body image concerns and did not have an internal weight target. She denied any history of excessive exercise, purging or use of laxatives.

Family denied any history of developmental concerns and she denied any academic or social difficulties in school or at home. The patient and her family denied any history of social communication difficulties, restrictive or repetitive behaviours. She denied low mood or concerns of anxiety.

### Investigations

**Fig. 3:**
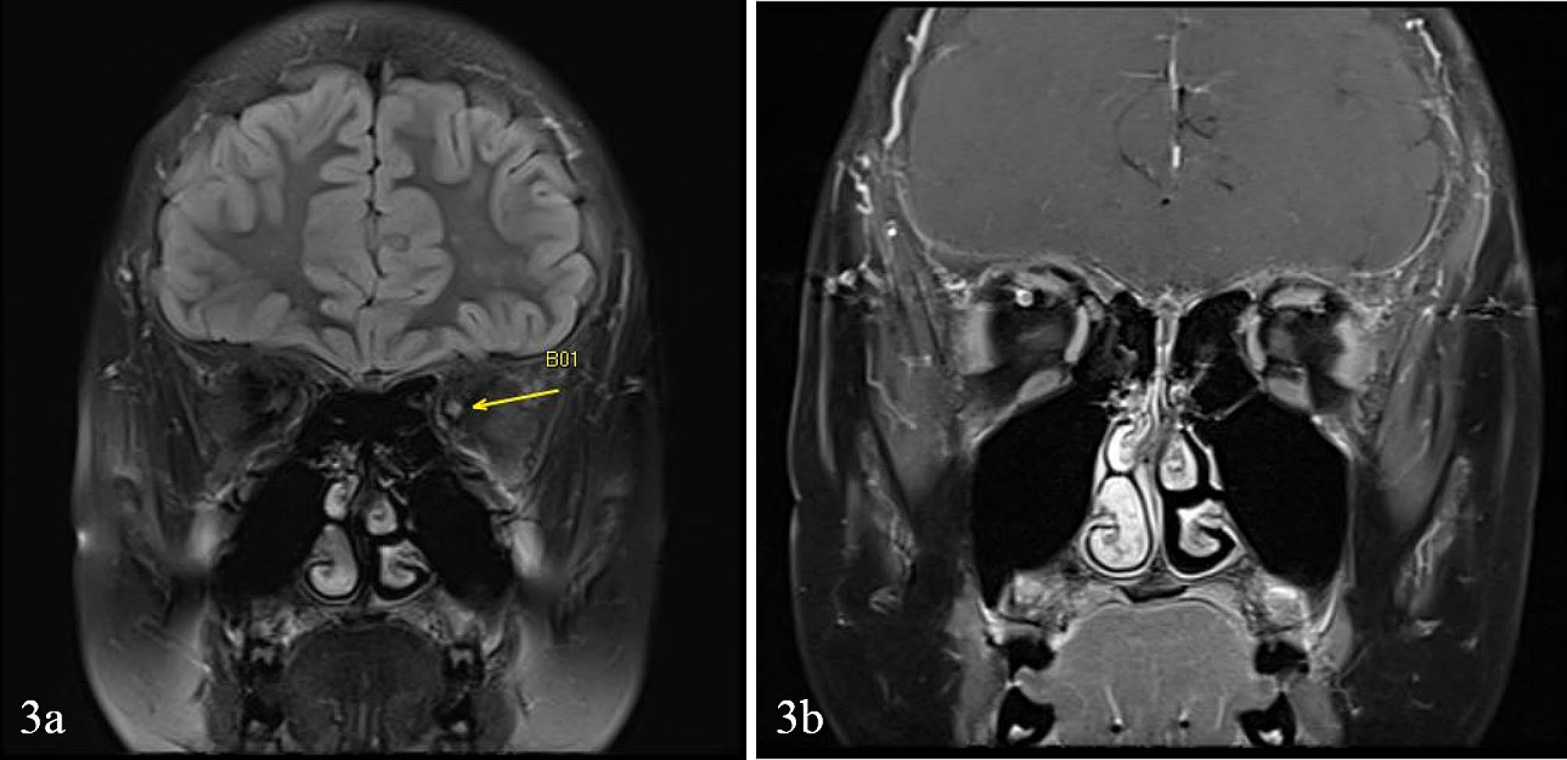
MRI imaging showing increased T2 signal intensity in the intra-orbital segment of the left optic nerve (Fig. 3**a**), with minimal enhancement noted on post-contrast T1 sequences (Fig. 3**b**)

Magnetic Resonance Imaging of the brain and orbits demonstrated increased signal with minimal enhancement in the left optic nerve, suggestive of left optic neuropathy (Fig. 3). A lumbar puncture was done with no pleocytosis and negative CSF cultures. Full blood count showed macrocytosis with a normal haemoglobin level, and liver function test was normal apart from mild hypoalbuminemia. Further investigations were sent to evaluate for micronutrient deficiencies that can cause optic neuropathies, along with autoimmune and metabolic studies.

There were significantly low serum levels of Vitamin A (< 0.02 mg/L, normal range 0.26–0.49), serum Vitamin B9 (folate) (8.2nmol/L, normal range > 26), red blood cell (RBC) folate (436nmol/L, normal range > 829) and Vitamin D (7.4ng/mL, normal range 20–100). Serum Vitamin B12 (cobalamin) (182pmol/L, normal range 186–830) and copper were mildly low (605 ul/L, normal range 734–1714). Zinc and other micronutrient levels were normal (Table 1).

**Table 1:** Results of micronutrient testing

	Result	Normal range
Vitamin A	**< 0.02 mg/L**	0.26–0.49 mg/L
Vitamin B1 (thiamine)	187 nmol/L	70–180 nmol/L
Vitamin B2 (riboflavin)	15 mcg	1–19 mcg
Vitamin B3 (nicotinamide)	31.5 ng/mL	5–48 ng/mL
Vitamin B5 (pantothenic acid)	99.46 ug/L	37–147 ug/L
Vitamin B6 (pyroxidone)		
Pyridoxal 5-phopshate Pyridoxic acid	21 mcg/L 3 mg/L	5–50 mcg/L 3-30 mg/L
Vitamin B7 (biotin)	229.8 pg/mL	100-2460.2 pg/mL
Vitamin B9 (folate), serum	**8.2 nmol/L**	> 26 nmol/L
Vitamin B9 (folate), RBC	**436nmol/L**	> 829 nmol/L
Vitamin B12 (cobalamin)	**182 pmol/L**	186–830 pmol/L
Vitamin D	**7.4 ng/mL**	20–100 ng/mL
Vitamin E	8.8 mg/L	5.5-9.0 mg/L
Calcium (adjusted by albumin)	2.44 mmol/L	2.30–2.63 mmol/L
Phosphate	1.9 mmol/L	1.3–1.9 mmol/L
Magnesium	1.00 mmol/L	0.86-1.17mmol/L
Copper	**605 ul/L**	734–1714 ug/L
Zinc	798 ug/L	724–1244 ug/L

### Clinical progress and outcomes

Based on her history, she was started on Vitamins A, B12, folic acid and thiamine supplements empirically. In consultation with the Neurology and Neuroopthalmology specialty teams, IV methylprednisolone was started empirically to treat for potential optic neuritis but was discontinued when investigations confirmed the diagnosis of a nutritional optic neuropathy. A urinalysis revealed pyuria, and urine culture done was consistent with a urinary tract infection and she was treated with a course of oral antibiotics.

Dietitian review noted inadequate intake of vitamins and minerals including calcium, zinc, Vitamin A, and Vitamin D related to limited food variety as evidenced by no regular intake of meat and alternatives, milk and dairy fruit and vegetables. She was started on maintenance multivitamin and calcium supplements, along with replacement doses of vitamin A, vitamin B12, folate, and Vitamin D. She had no evidence of refeeding syndrome or medical instability due to malnutrition. She was evaluated by the Eating Disorder Service who felt her presentation was consistent with ARFID. The patient and family received extensive dietary counselling and psychoeducation.

Multidisciplinary outpatient follow-ups were arranged. On review at 2 months from discharge, weight had increased from 36 kg to 40.2 kg. By 4 months post discharge, repeat serum folate, RBC folate, Vitamin B12, Vitamin D and copper levels were replete.

Visual acuity had improved to 6/6 in the right eye and 6/18 in the left eye. She had a residual left optic neuropathy as evidenced by a left relative afferent pupillary defect, decreased visual acuity, impaired colour vision (Hardy Rand Rittler plates 10/10 on the right and 3/10 on the left) and a residual centrocaecal scotoma on the left eye visual field.

While she had continued to have difficulties eating many vegetables and fruits, she had started to eat a wider range of foods prepared by her family. She was adherent to her multivitamin supplementation and parents noted minimal resistance to food prepared by her family.

## Discussion

This case illustrates an example of micronutrient deficiency that resulted from ARFID, which presented uniquely with visual complaints.

### Diagnosis of ARFID

Our patient met DSM-5 diagnostic criteria for ARFID [1]. Her disordered eating, with restricted food choices and avoidance of specific food groups, resulted in significant nutritional deficiencies including that of Vitamin A, folate, Vitamin B12, copper and Vitamin D, manifesting in visual loss. While there were initial concerns of anorexia nervosa, as her food variety had become increasingly restricted after comments regarding her weight, she had not engaged in significant caloric restriction. On further probing, she denied an intense fear of gaining weight and significant body image disturbances. She had continued to take caloric-dense foods such as bread with sweetened nut spread, with no compensatory purging or exercise. Her selective eating had also started from a young age, which is in keeping with a diagnosis of ARFID [2].

### Nutritional deficiencies in restrictive eating disorders

Individuals with restrictive eating disorders such as ARFID and anorexia nervosa can have significant deficiencies in macro- and micronutrient intake, with a risk of developmental and health problems. A dietary review of children with ARFID by Schmidt et al. [3] found significantly lower total energy and protein intake in children with ARFID versus controls, with significantly reduced variety of food intake in all food groups except carbohydrates. Those with ARFID met only 20–30% of the recommended intake for most vitamins and minerals, with significantly lower intake relative to controls for Vitamin B1, B2, C, K, zinc, iron, and potassium. Similarly, patients with anorexia nervosa are at risk for micronutrient deficiencies. A retrospective review of adult patients with anorexia on follow up in an outpatient clinic noted vitamin deficiencies in 45.7% of patients [4]. The incidence is higher in those with significant malnutrition. In a large cohort of patients with anorexia hospitalised for anorexia nervosa, 92.8% of patients had at least one micronutrient deficiency [5]. The most prevalent micronutrient deficiencies were in zinc, Vitamin D, copper, selenium, Vitamin B1, B12 and B9.

Our patient had a significant deficiency in Vitamin A, both biochemically and clinically. Whilst more commonly seen in low- and middle-income countries, it is increasing seen in high-income countries as a result of chronic conditions such as chronic liver disease or other causes of gastrointestinal malabsorption [6]. Vitamin A, the most active form, can be found in animal sources such as liver, kidney, egg yolk and butter; whereas provitamin A is found in plant sources such as leafy vegetables, sweet potatoes and carrots.

In the eye, Vitamin A is crucial to cellular differentiation and integrity, with deficiency resulting in xeropthalmia with dryness and clouding of the cornea. It also has a key role in phototransduction, with deficiency causing night blindness [7]. Extra-ocularly, Vitamin A is critical in mucosal epithelial regeneration and immune function, and deficiencies can cause immune system impairment. Dermatologic manifestations include follicular hyperkeratosis, and together with Vitamin D deficiency [8] can cause worsening eczema as in our patient. Vitamin A deficiency can also cause poor bone growth and osteoporosis. Finally, infectious diseases can precipitate Vitamin A deficiency by decreasing intake and absorption, and increasing excretion [9]. In this case, our patient was noted to have a urinary tract infection which may have precipitated her acute presentation.

Diagnosis is made by clinical findings, with treatment for xerophthalmia given empirically as in this case and can be confirmed by measurement of serum Vitamin A levels. There is one existing case report of Vitamin A deficiency in a patient with ARFID, who was admitted for malnutrition with noted gradual blurring of vision [10]. In a systematic review of children with autism spectrum disorder with severe self-imposed dietary restrictions, Vitamin A deficiency was the second-most common nutrient deficiency disease (17.1% of cases), after Vitamin C deficiency [11]. In some cases, this resulted in permanent visual loss.

Vitamin A deficiency in itself is not known to cause optic neuropathy. Vitamin B1 (thiamine), B6, B9 (folate), B12, copper and zinc have been implicated in optic neuropathy, most common in adult patients with alcoholism or after bariatric surgery [11–14]. This can present in a varied fashion, with either unilateral or bilateral loss and with acute, subacute, or slowly progressive presentation. There have been case reports of acute visual loss from optic neuropathy from thiamine deficiency resulting from gastrointestinal illnesses with increased losses and reduced oral intake [15]. Patients with eating disorders are at risk of thiamine deficiency, with a review of food diaries of patients with ARFID found that patients took a mean of 19.1% of the daily recommended intake of thiamine [3]. Thiamine deficiency was detected in 6% of patients on their first admission for eating disorder [16]. In our patient, the severely low levels of serum and RBC folate is the most likely cause of her optic neuropathy, although Vitamin B12, and copper were also mildly low. Extensive workup for other causes were negative. There was also clinical improvement after appropriate vitamin replacement.

## Conclusion

This case highlights a unique presentation of a patient with ARFID, with visual loss secondary to Vitamin A and folate deficiency. While other medical causes must be ruled out in patients with visual loss, characteristic findings on ophthalmological examination and blood tests suggested nutritional deficiency as the cause of her visual loss, and further evaluation confirmed the diagnosis of ARFID. Eating disorders should be considered as a cause of micronutrient deficiencies, even in the absence of weight loss. Similarly, micronutrient deficiencies should be considered in appropriate cases of visual changes and a dietary history should be screened. Providers should be aware of the increased risk of micronutrient deficiencies in children and consider the need for appropriate testing and supplementation based on clinical findings.

## Data Availability

All data generated or analysed during this study are included in this published article.

## Abbreviations

ARFID: avoidant/restrictive food intake disorder
